# A gold standard set of mechanistically diverse enzyme superfamilies

**DOI:** 10.1186/gb-2006-7-1-r8

**Published:** 2006-01-31

**Authors:** Shoshana D Brown, John A Gerlt, Jennifer L Seffernick, Patricia C Babbitt

**Affiliations:** 1 Department of Biopharmaceutical Sciences, University of California, 1700 4th Street, San Francisco, San Francisco, CA 94143-2550, USA; 2 Department of Biochemistry, University of Illinois, Roger Adams Laboratory, 600 S Mathews Avenue, Urbana, IL 61801, USA; 3 Department of Biochemistry, Molecular Biology, and Biophysics, Biological Process Technology Institute, and Center for Microbial and Plant Genomics, University of Minnesota, St Paul, MN 55108, USA; 4 Departments of Biopharmaceutical Sciences and Pharmaceutical Chemistry, University of California, 1700 4th Street, San Francisco, San Francisco, CA 94143-2550, USA

## Abstract

Superfamily and family analyses provide an effective tool for the functional classification of proteins, but must be automated for use on large datasets. We describe a 'gold standard' set of enzyme superfamilies, clustered according to specific sequence, structure, and functional criteria, for use in the validation of family and superfamily clustering methods. The gold standard set represents four fold classes and differing clustering difficulties, and includes five superfamilies, 91 families, 4,887 sequences and 282 structures.

## Abstract 2

A gold standard set of enzyme superfamilies, clustered according to sequence, structure and functional criteria, is presented.

## Background

With large volumes of sequence and structural data now available, functional characterization of proteins has become the rate-limiting step in putting biological information to practical use. Large-scale functional annotation efforts have focused on automated strategies, as more traditional methods, such as experimental characterization of gene function and manually curated analysis of gene sequence and structure, can only be used efficiently on small subsets of the available data.

While this scale-up of the analysis process is required to handle the sheer volume of new information, automated analysis strategies possess inherent and serious limitations. For example, simple pairwise comparisons have been shown to be inadequate for functional classification of proteins with less than 30% to 40% identity [1-3]. Utilizing information from multiple related sequences, especially via probabilistic methods such as sequence profiles or hidden Markov models [4-6], the number of true evolutionary relationships found between proteins with less than 30% identity can be tripled [1,3]. Unfortunately, even when true homologous relationships are detected, direct transfer of functional annotation is not often possible at low levels of sequence identity [2,7-9].

Even when direct transfer of the full functional annotation is not possible, evolutionarily related proteins usually share some functional relationship. To determine what this relationship is, we must start by examining the type of evolutionary linkage between the proteins. Here we have concentrated on enzymes because they have a well-defined biochemical function - the catalysis of a particular reaction.

Horowitz suggested that ligand binding is the dominant constraint guiding enzyme evolution [10,11]. According to his theory, biochemical pathways evolved backwards. When the substrate for the final enzyme in the pathway was depleted, a new enzyme evolved from this enzyme, via gene duplication and divergence, to produce the needed substrate from an available precursor. While the reaction mechanism of the new enzyme was allowed to drift away from that of the original enzyme, the ability to bind the common substrate/product was retained. Although this theory appears to apply to some groups of enzymes, for example HisA/HisF in the histidine biosynthesis pathway and TrpF/TrpC in the tryptophan biosynthesis pathway [12], it does not appear to be the dominant mechanism governing enzyme evolution [13]. Furthermore, the model typically applies only to pairs of divergent enzymes.

Chemistry-driven evolution [14-16], an alternative theory that appears to represent a substantial proportion of enzymes [13], identifies a chemical step or capability as the dominant constraint guiding enzyme evolution. According to this model, a newly evolved enzyme retains a fundamental chemical capability of its progenitor. The newly evolved enzyme may catalyze a reaction similar to its progenitor with only an altered substrate specificity, or it may catalyze a quite different overall reaction while still retaining some chemical capability common to its progenitor [12].

A group of related enzymes that share a common chemical capability mediated by conserved catalytic elements but catalyze different overall reactions has been termed a mechanistically diverse superfamily [12]. A mechanistically diverse superfamily can be subdivided into families, where a family is defined as a group of related enzymes whose members catalyze the same overall reaction via conserved catalytic elements. Each of these mechanistically diverse superfamilies may contain hundreds or even thousands of proteins, representing many different overall functions and utilizing a wide range of substrates.

Mechanistically diverse superfamilies pose an especially difficult problem for automated functional classification methods due to the complexity of their underlying biology. For example, a newly sequenced superfamily member may not catalyze the same overall reaction as its closest relative in the superfamily, but may instead be related to other superfamily members by a more subtle conserved chemical capability. If the superfamily itself has not been characterized, the conserved chemical capability may not be immediately obvious. It is thus useful to subdivide a superfamily into families containing enzymes that catalyze the same overall reaction.

Sequence and structural similarity alone cannot be used to cluster sequences into families because different families evolve at different rates [17] (M.E. Glasner, R.A. Chiang, N. Fayazmanesh, M.P. Jacobsen, J.A.G, P.C.B., unpublished data; J.L.S., L.P. Wackett, P.C.B. unpublished data). Consequently, the boundaries between different families within a superfamily are uneven in sequence and structure space; in some cases, even very highly similar sequences may perform different reactions. In the mechanistically diverse amidohydrolase superfamily, for example, melamine deaminase and atrazine chlorohydrolase share 98% sequence identity, but catalyze different reactions [18].

Likewise, functional information alone cannot be used to cluster proteins into superfamilies and families, due to convergent evolution, in which nature has evolved more than one structural strategy to perform a given chemical reaction [19-21]. For example, George *et al*. [21] found that 69% of the functions described by three digit EC numbers are found in multiple Structural Classification of Proteins database (SCOP) [22] superfamilies, suggesting, at least for some of these, independent evolutionary origins. Further, some functions are found in multiple SCOP fold classes, providing further evidence that they have evolved via convergent evolution [20,21]. Thus, although enzymes in these groups catalyze the same overall reaction, they likely utilize different mechanisms.

Even within a single superfamily, the same function may have evolved more than once [23]. For example, the ability to hydrolyze an organophosphate appears to have evolved on at least two separate occasions within the common lineage of the amidohydrolase superfamily (J.L.S., L.P. Wackett, P.C.B., unpublished data). The distinct evolutionary origins of the aryldialkylphosphatase family and the phosphotriesterase family are reflected in an extremely low overall sequence identity between the two families and by subtle differences in the constellation of active site residues used to catalyze the common reaction.

To address these issues and provide a useful test set for benchmarking and development of tools for functional inference, we have constructed a new gold standard set of mechanistically diverse enzyme superfamilies. Most importantly, these proteins are clustered according to rigorous and systematic definitions of family and superfamily. Because these definitions map specific elements of protein sequence and structure to specific elements of function, gold standard families and superfamilies are especially useful for developing tools for elucidation of function of uncharacterized members. Moreover, because they represent related proteins whose functions have diverged, sometimes substantially, they may serve as a challenging test set for automated superfamily clustering methods based on either sequence or structure. To further enhance the utility of the gold standard set as a test set for evaluation of automated superfamily clustering methodologies, evidence codes, based on those developed by the Gene Ontology consortium [24], are provided for all functional assignments.

## Results

As of August 2005, our five gold standard superfamilies include four distinct fold classes and contain a total of 91 families, 4,887 sequences and 282 structures (Table 1). For the purposes of this paper, we have defined two different types of families. Gold standard families contain only sequences with either experimentally determined functions or sequences that are highly similar to them, that is, show highly significant BLAST e-values (≤ 1 × 10^-175^) to experimentally characterized sequences. In addition, each of the sequences in a gold standard family is required to conserve all family-specific catalytic residues identified from the literature. Silver standard families contain all the sequences from the corresponding gold standard family, but may also contain additional sequences that have not been experimentally characterized, show an e-value between 1 × 10^-20 ^and 1 × 10^-175 ^to a characterized family member, and meet other relevant criteria (see Materials and methods).

Table 2 gives a detailed view of the gold and silver standard families that make up each superfamily. As shown in this table, these families catalyze a wide variety of reactions, spanning five of the six EC classes. The superfamily sequence sets represent different diversity levels, as described further in the Discussion. All of the gold standard superfamilies have been rigorously studied, and their structure-function relationships extensively interpreted, providing detailed information, including reaction mechanisms, superfamily-specific catalytic residues, and family-specific catalytic residues (see J.L.S., L.P. Wackett, P.C.B., unpublished data, and [25-36] and references therein, for reviews and general descriptions of these superfamilies.) We have compiled this information (as well as information on additional superfamilies) into a publicly available database that explicitly links enzyme sequence, structure and function in the manner described above [37-39]. (Structure-Function Linkage Database (SFLD) superfamilies correspond to gold standard superfamilies in this paper. SFLD families correspond to the silver standard families in this paper.)

### Comparison of gold and silver standard superfamilies and families to existing classifications

We compared the family and superfamily classifications of the sequences in all five of our superfamilies to that of the Protein Families database (Pfam) [40] (families only), SCOP (families and superfamilies) and SUPERFAMILY [41] (a set of hidden Markov models based on SCOP superfamilies) databases. Additional data file 1 shows the difference between our family and superfamily classifications and those of Pfam, SCOP and SUPERFAMILY, for each individual sequence in our five superfamilies.

**Figure 1:**
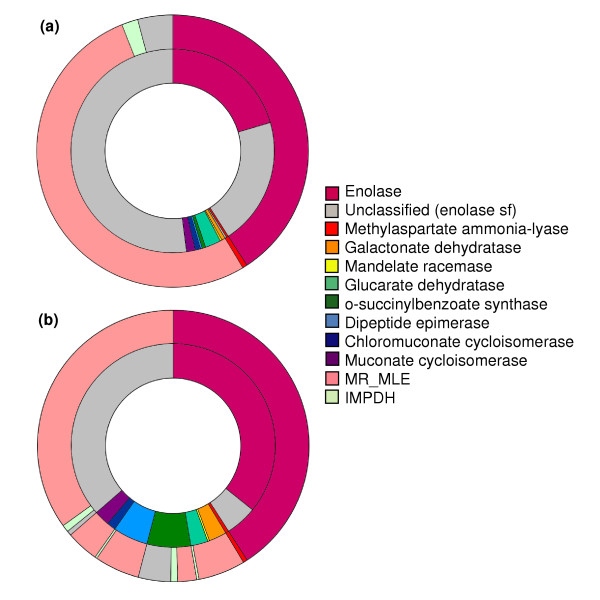
Comparison of gold and silver standard family classifications to Pfam for the gold standard enolase superfamily. The outer ring represents Pfam family classifications. Sequences that match multiple Pfam HMMs, all of which correspond to a single SFLD functional domain (for example, 'Enolase_N', representing the amino terminus of the enzyme enolase and 'Enolase', representing the carboxyl terminus of the enzyme enolase), are shown with a single designation in the figure to simplify the illustration. **(a) **The inner ring represents gold standard family classifications. Gray regions represent enzymes that can be assigned to the gold standard enolase superfamily, but cannot be confidently assigned to a gold standard family. **(b) **The inner ring represents silver standard family classifications. Gray regions represent enzymes that can be assigned to the gold standard enolase superfamily, but cannot be confidently assigned to a silver standard family.

The main difference between our family classifications and those of Pfam and SCOP is their coverage of function space. As shown in Table 3, our gold and silver standard families include only sequences that catalyze a single overall reaction. Although some SCOP and Pfam families (for example, the enolase family) correspond to this level of functional similarity, Table 3 shows that most are broader, principally because these classification systems rely mainly on overall sequence and structural similarities rather than on the finer granularity analysis focused on the subsets of catalytic residues that distinguish enzymes that perform a specific catalytic reaction. For example, the Pfam MR_MLE_N and MR_MLE families include enzymes that catalyze at least seven different overall reactions. This difference is illustrated graphically in Figure 1.

Figure 1 also shows that some of the enzymes in our gold standard enolase superfamily are classified into the Pfam IMPDH family, which contains inosine monophosphate dehydrogenases, among other enzymes. Although the members of the IMPDH family share the (β/α)_8 _(TIM) barrel fold common to enolase superfamily members, they do not have the amino-terminal domain found in all enolase superfamily members, nor do they use a similar set of catalytic residues to perform their functions. Thus, we believe that classification of any enolase superfamily members into the Pfam IMPDH superfamily is incorrect.

Superfamily classifications for four of our five gold standard superfamilies (amidohydrolase, enolase, haloacid dehalogenase, and vicinal oxygen chelate) correspond to the analogous SCOP and SUPERFAMILY superfamily designations. In contrast, the gold standard crotonase superfamily is only a subset of the corresponding Clp/crotonase superfamily in SCOP and SUPERFAMILY. The SCOP Crotonase-like family contains enzymes corresponding to the gold standard crotonase superfamily, while the remaining families listed in the SCOP Clp/crotonase superfamily contain enzymes that may be evolutionarily related to gold standard crotonase superfamily members, but do not have an established mechanistic linkage [42,43]. Again, because there is no explicit indication of the functional similarity contained within a SCOP or SUPERFAMILY superfamily, it is difficult to use these classifications to make functional inferences regarding uncharacterized proteins.

## Discussion

### Diversity of gold standard superfamilies

The five gold standard superfamilies contain enzymes exhibiting varying levels of sequence diversity. On one end of the spectrum, the enolase and crotonase superfamilies contain a rather discrete set of sequences, such that most of their constituent families exhibit statistically significant levels of sequence similarity to other superfamily members. On the other end of the spectrum are the haloacid dehalogenase superfamily and some branches of the amidohydrolase superfamily, which contain the most diverse sets of sequences, including a high proportion of outlier sequences that have only low levels of sequence identity to their closest superfamily relative(s). Because it provides a set of superfamilies with a range of sequence diversity, the gold standard set is a useful (and challenging) test set for automated methods designed to collect and cluster sequences by function.

The superfamilies in the gold standard set are not the only mechanistically diverse superfamilies found in nature. Additional mechanistically diverse superfamilies are described in the SFLD and in other work (see [12] for some examples), and perhaps many more uncharacterized superfamilies are likely to exist. Although no current research provides an adequate count of mechanistically diverse superfamilies, some rough estimates can be made. For example, of the 339 superfamilies listed in the SCOPEC database, 49% contain two or more families with differences in EC number at all four positions [21]. This suggests, for the enzyme superfamilies that have been catalogued in SCOPEC, a rough upper bound on the possible number of mechanistically diverse superfamilies that include at least two different overall reactions. But because the identification of a mechanistically diverse superfamily requires an understanding of the underlying mechanism of the member enzymes, it is difficult to estimate the total number of such superfamilies found in nature. The gold standard superfamilies described in this work represent the best characterized subset of mechanistically diverse superfamilies for which we have a large amount of functional and mechanistic information and that have thus far been added to our SFLD.

### How do gold standard family and superfamily classifications differ from those of existing databases such as SCOP and Pfam?

Pfam, SCOP, and other similar databases have become the standards by which new tools for functional and evolutionary classification of protein sequences are validated [44-47]. (Additional test sets, such as BAliBASE [48] and SABmark [49], are designed to evaluate new sequence alignment methods rather than superfamily or family clustering algorithms.) We compare our family and superfamily classifications to those found in Pfam, SCOP, and SUPERFAMILY (a set of hidden Markov models based on SCOP superfamilies) to demonstrate the unique properties of our classifications compared to these standards.

#### Structural domains versus functional domains

The SCOP database classifies all proteins into structural domains. Pfam also uses structural information, where available, to ensure that families correspond to a single structural domain. In contrast, we have used both structure and function-based definitions to divide proteins into their component domains. For example, SCOP and Pfam divide the enzymes in the enolase superfamily into amino-terminal and carboxy-terminal structural domains. However, because the amino- and carboxy-terminal structural domains are both required for functionality, we have kept these sequences as a single functional domain.

In keeping with our function-based domain definition, when a protein contains two or more distinct active sites, we subdivide the protein into separate functional domains, each containing a single active site, if they occur as separate proteins in other species. These functional domains are then classified by family and superfamily.

### Does sequence and structural conservation imply functional conservation?

Specific molecular function - defined here as the overall reaction catalyzed by an enzyme - is often not conserved across a group of related enzymes, particularly in mechanistically diverse enzyme superfamilies. Although early studies suggested that above 40% identity all four digits of an EC number (which specifies a single overall reaction) are conserved between enzyme-enzyme pairs [2], later studies that correct for database bias have challenged these conclusions. Burkhard Rost, for example, reports that less than 30% of enzyme-enzyme pairs above 50% identity have entirely identical EC numbers [8], and Tian and Skolnick report that pairwise sequence identity of at least 60% is required to transfer all four digits of an EC number with 90% accuracy [7]. Thus, it is not surprising that most of the SCOP and Pfam families corresponding to our gold standard superfamilies contain enzymes that catalyze more than one overall reaction (Table 3 and Figure 1).

But while specific molecular function may not be conserved across a group of related enzymes, some aspect of molecular function is often conserved. For example, Tian and Skolnick report that pairwise sequence identity of at least 60% is required to transfer all four digits of an EC number with 90% accuracy [7]. Furthermore, because the EC system was not designed to capture mechanistic information about the reaction in question [9], enzyme-enzyme pairs with completely different EC numbers may still share some aspect of function [20].

Our gold standard superfamilies have been designed with exactly this type of functional similarity in mind. Not only are enzymes in a gold standard superfamily thought to be evolutionarily related based on sequence and structural criteria, they also share a set of catalytic residues thought to be responsible for a common chemical capability. This common capability may be a mechanistic step (for example, abstraction of a proton alpha to a carboxylic acid to form an enolate anion intermediate in the enolase superfamily), or a structural strategy for stabilizing a common intermediate (for example, use of an oxyanion hole to stabilize an enolate anion intermediate derived from the acyl-CoA ester derivatized compounds that are substrates in the crotonase superfamily). In each superfamily, the cognate chemical capability is mapped to specific amino acids, thus allowing uncharacterized proteins identified as candidate superfamily members to be evaluated for their ability to perform the superfamily-specific chemistry based on the presence or absence of this amino acid signature.

The division of gold standard superfamilies into families again utilizes sequence, structure and functional information. Not only do the enzymes in a family form a more closely related subset, based on their sequences and structures, compared to the rest of the superfamily, they are also thought to catalyze a single overall reaction. Because the overall reaction has been mapped to a common set of catalytic amino acids shared by all family members, uncharacterized proteins can be evaluated for their ability to perform the family-specific reaction based both on overall sequence or structural similarity to family members and on the presence of the active site motif. These family-specific motifs can be used as part of a system to differentiate families within a given superfamily, as many of the family-specific motifs contain family-specific residues in addition to the superfamily-specific catalytic residues. (In fact, a recent study has demonstrated the importance of using catalytic residue information to identify proteins that are functionally related, showing that the inclusion of such information improves the accuracy of annotation transfer, especially between distantly related proteins [50].)

In contrast, the level of functional similarity required to classify a sequence according to SCOP, SUPERFAMILY, or Pfam is not uniform. While some SCOP and Pfam families consist of enzymes that catalyze the same overall reaction, many encompass enzymes catalyzing several reactions (Table 3 and Figure 1). Likewise, the level of functional similarity shared between enzymes in a SCOP or SUPERFAMILY superfamily is not uniform (see Results). Because there is no specific indication of the level of functional similarity shared by sequences in a SCOP, SUPERFAMILY, or Pfam grouping and no mapping of conserved functional elements to conserved sequence or structural elements, there is no simple and systematic way to use these classifications to infer the specific molecular function of an uncharacterized enzyme. Additional family and superfamily classifications [51-54], as well as automated methods designed to cluster proteins into superfamilies and families [41,45,47], suffer from similar problems. These databases and methods are valuable resources, but they may not be the right tools to use for all purposes. In particular, when functional classification of divergent enzymes is a goal, our gold standard families and superfamilies may serve as a more appropriate test set.

### Complications for functional inference in mechanistically diverse superfamilies

In the development of the gold standard set, we encountered several difficulties in attempting to classify sequences that belong to mechanistically diverse superfamilies into their constituent families. These difficulties largely arise from the complexity of the underlying biology, where the boundaries between different families within a superfamily may be uneven due to different evolutionary rates within each family, and, due to a number of reasons, some enzymes may not fit into the simple family classification at all.

For example, although the gold and silver families provided here represent a large number of different reactions evolved along each superfamily lineage, these proteins by no means represent all sequences that can be included in the associated superfamilies. Because annotation transfer for distantly related sequences in mechanistically diverse superfamilies is not trivial, we have not included sequences in either the gold or silver standard family sets unless they meet the stringent criteria defined in the Methods section. Thus, Figure 1 shows that some of the enzymes in our gold standard superfamilies have not been assigned to a family (gray areas on the inner rings), even though we can confidently assign them to a superfamily based on their overall sequence or structural similarities and the conservation of active site residues associated with the canonical superfamily partial reaction or chemical capability. In some cases, this incomplete classification is due to the fact that the family-specific overall reaction has not yet been identified. In other cases, while there may be some evidence to suggest that the enzyme in question belongs to one of the existing families, it is so distantly related in sequence that it cannot be confidently assigned to the family without additional data such as further mechanistic characterization or tertiary structural information. As a result, sequences that fall into the gray areas of the inner rings in Figure 1 are not included in the gold or silver family sets. It is not uncommon for half the enzymes in a gold standard superfamily to lack a family assignment.

Even when our stringent criteria for family classification are used, we cannot be absolutely certain enzymes that have not been experimentally characterized are correctly classified. For example, the enzymes melamine deaminase and atrazine chlorohydrolase from *Pseudomonas *are 98% identical, but catalyze different overall reactions within the amidohydrolase superfamily [18]. The two enzymes are classified into separate families within our gold standard set; however, if experimental data had not been available to distinguish the two functions of these highly similar enzymes, we would likely have classified both enzymes into the same family due to their high sequence identity and conservation of known catalytic residues. Although such a high degree of sequence similarity coupled with functional divergence is not common [2,7,8], it is certainly possible that other such examples could exist in our gold standard set. Family boundaries are thus expected to change slightly as additional experimental information becomes available. Updated versions of our gold and silver standard families will, therefore, be made available on the SFLD website [38] as new information warrants.

An additional difficulty for the subclassification of superfamily enzymes into families is the somewhat arbitrary assumption we make that all enzymes in a given family catalyze a single biologically relevant overall reaction. In reality, some enzymes may have evolved to be nonspecific, for example, the cytochrome P450s, which are involved in the metabolism of a wide variety of endogenous and exogenous toxins. In addition to this rather extreme example, many enzymes can turn over multiple related substrates at varying levels of proficiency. In some cases, such promiscuity is biologically relevant, while in other cases, it may only be seen *in vitro*. In either case, this complicates the family classification process. For example, the extradiol dioxygenase enzymes within the vicinal oxygen chelate superfamily are difficult to subclassify into families because they are similar in sequence and utilize a common set of active site residues due to their similar chemistry. Further complicating this is the fact that many of these enzymes have been shown to catalyze the extradiol cleavage of several related substrates, and it is not always clear which substrate is biologically relevant. We have noted those families that are especially difficult to classify in the footnotes to Additional data files 1 and 2.

Despite such complications, in many cases we can find clear boundaries between functionally distinct families. In these cases, subclassification of a superfamily into families facilitates the process of making functional inferences about uncharacterized proteins.

## Conclusion

We have described a gold standard set of proteins, clustered according to systematic and consistent definitions of family and superfamily. Because these definitions map specific elements of protein sequence and structure to specific elements of function, gold standard families and superfamilies are optimized for use in elucidation of the function of uncharacterized members, and serve as a new type of test set for automated superfamily clustering methods. The opportunities this test set provides to aid in detailed validation of such clustering methods will contribute to advances in automated annotation of newly sequenced genomes.

## Materials and methods

### Definitions and requirements for gold standard superfamilies and families

We define a mechanistically diverse enzyme superfamily as a group of homologous enzymes that catalyze different overall reactions via a common mechanistic attribute that requires conserved catalytic elements. We define a family as a subset of a superfamily where all enzymes catalyze the same overall reaction via the same mechanism.

Prior to addition of a superfamily to our gold standard set, we ensure that the following conditions are met. Firstly, crystal structures for proteins from at least two different families within the superfamily are available. Secondly, sufficient mechanistic information for proteins from at least two different families within the superfamily are available, thus allowing the common partial reaction or chemical capability to be identified. Thirdly, experimental evidence regarding the identity of catalytic residues involved in the conserved partial reaction or chemical capability is available for sequences in at least two different families.

### Semi-automated collection of superfamily sequences

We roughly based our sequence collection protocol on that outlined by Todd *et al*. [2] but used our own superfamily definitions, rather than those contained in the CATH database, to guide superfamily creation. For each family within a superfamily, we chose a sequence that had been shown experimentally to catalyze the family-specific reaction to serve as a query for PSI-BLAST [6]. Each PSI-BLAST analysis was performed against the National Center for Biotechnology Information nonredundant protein database at an expectation value cutoff of 5 × 10^-4 ^for 20 rounds or until convergence. All PSI-BLAST hits that aligned over at least 80% of the length of the query sequence were added to the superfamily of the query sequence.

### Manual inspection of superfamily sequences

Sequences collected via the automated protocol were inspected to verify superfamily membership by examining multiple sequence alignments for the presence of known catalytic residues and other superfamily specific sequence motifs

### Semi-automated clustering of superfamily sequences into families

Superfamily sequences were classified into families according to a two-step procedure. First, sequences were roughly clustered based on sequence similarity. Functional information from the literature was then used to refine family clusters.

Two types of family clusters were constructed, at different levels of stringency. Gold standard families contain sequences with experimentally determined functions (see below) and sequences that show highly significant BLAST e-values (≤ 1 × 10^-175^) to experimentally characterized sequences. In addition, each of the sequences in a gold standard family is required to conserve all family-specific catalytic residues identified from the literature. Silver standard families contain all the sequences from the corresponding gold standard family, but may also contain additional sequences that have not been experimentally characterized and show an e-value between 1 × 10^-20 ^and 1 × 10^-175 ^to a characterized family member. (In most cases, the e-value is much more significant than 1 × 10^-20^.) These additional sequences do, however, conserve all family-specific catalytic residues identified in the literature, and curators have used other information, such as examination of the sequences in the context of a family alignment and examination of operon context, to increase the confidence of these assignments.

### Experimentally characterized enzymes

For the purposes of family classification, enzymes with experimentally characterized function include enzymes that have been shown through a direct assay to catalyze a specific reaction or enzymes whose function has been inferred based on complementation or mutagenesis data. The literature references upon which each family classification was based can be found in Additional data file 5.

### Identification of family and superfamily-specific catalytic residues

We define catalytic residues similarly to Porter *et al*. [55]. We do not include residues that are described in the literature only as being involved in substrate binding, because these residues may not be as well conserved across a family as residues that play a more direct role in the catalytic mechanism of the enzyme (M.E. Glasner, R.A. Chiang, N. Fayazmanesh, M.P. Jacobson, J.A.G, P.C.B, unpublished data).

Following the criteria described above, family-specific catalytic residues were identified based on experimental data from the literature, including mutagenesis and X-ray crystallography data. When the literature contained catalytic residue information for multiple enzymes within a single family, the information was pooled and applied to the entire family. In some cases, experimental information regarding catalytic residues was not available for a given family, but catalytic residues could be inferred based on sequence similarity to related families, at least for the subset of catalytic residues involved in the partial reaction or chemical capability conserved across the superfamily. Superfamily-specific catalytic residues were identified by taking the subset of family-specific catalytic residues conserved across all enzymes in a superfamily that are involved in the partial reaction or chemical capability common to the superfamily. Generally, this approach has been validated for all of the superfamilies represented in this work, including homologous sequences in families for which no structures were yet available when these relationships were initially predicted. In several of these latter cases, experimentally determined structures have validated those inferences (see [15,56,57] for examples).

Although we made every effort to use our knowledge of the family and superfamily-specific chemistry to support homology-based catalytic residue prediction, this is to some extent a subjective process, and our family and superfamily-specific catalytic residue assignments may change as further experimental information becomes available. The type of evidence used to identify a given family or superfamily-specific catalytic residue may be determined by examining the associated evidence code in the SFLD, which is updated as new information about these superfamilies becomes available.

### Comparison of gold and silver standard families and superfamilies to existing classifications

To illustrate the differences between our family and superfamily classifications and existing classifications, we have compared our data to Pfam, SCOP and SUPERFAMILY (see Additional data files 1 and 2).

Each of the sequences in our superfamilies was compared to the global-alignment-based hidden Markov models contained in version 17.0 of the Pfam-A database [40], using HMMPFAM [58] with the gathering cutoff established by Pfam curators. Sequences were classified into the Pfam-A family to which they showed the most significant match. When a sequence corresponded to multiple Pfam domains, the most significant match for each region of the sequence was noted.

The SCOP family and superfamily classifications were obtained for each sequence in our superfamilies that had a crystal structure listed in SCOP version 1.67. Each of the sequences in our superfamilies was also compared to the SUPERFAMILY set of hidden Markov models [41], which were built based on SCOP release 1.67. Comparisons were performed using HMMPFAM, with an e-value cutoff of 1. Sequences were classified into the SUPERFAMILY superfamily to which they showed the most significant match. When a sequence corresponded to multiple SUPERFAMILY domains, the most significant match for each region of the sequence was noted.

## Additional data files

The following additional data are available with the online version of this paper. Additional data file 1 lists the family and superfamily mappings for the sequences and structures in the gold standard superfamily set, with Pfam, SCOP, and SUPERFAMILY assignments listed as names. Additional data file 2 lists family and superfamily mappings for the sequences and structures in the gold standard superfamily set, with Pfam and SCOP assignments listed as accession numbers. Additional data file 3 provides fasta format sequences for gold standard superfamily proteins. Additional data file 4 contains references for the gold and silver standard family assignments. Additional data file 5 lists gold and silver standard family assignments and the corresponding references.

## Supplementary Material

Additional data file 1 Superfamily and family assignments for each of the sequences and structures from this work, as well as the corresponding Pfam, SCOP, and SUPERFAMILY assignments. Pfam, SCOP, and SUPERFAMILY assignments are listed as names. ^1^National Center for Biotechnology Information GI number. Additional data file 3 contains the fasta format sequences corresponding to each gi number. ^2^Protein Data Bank identifier. ^3^The gold and silver standard *o *-succinylbenzoate synthase (OSBS) families contain a more diverse set of enzymes than many other families listed in the table. All of the OSBS enzymes are believed to catalyze the same overall reaction via the same catalytic residues and there is no convincing evidence to suggest convergent evolution from within the superfamily, so we believe that these enzymes meet our definition of family. They appear, however, to utilize a different constellation of substrate binding residues, and certain subclusters within the family catalyze the promiscuous N-acyl amino acid racemase reaction in addition to the OSBS reaction. Because the sequences that comprise this family are highly divergent, it may pose special difficulties for automated clustering methods. Additional families that may be especially challenging include the extradiol dioxygenase families within the VOC superfamily, where a relatively high degree of sequence similarity and catalytic promiscuity make accurate clustering difficult. ^4^Evidence code for gold and silver standard family assignment [59]. ^5^ID number for the literature reference upon which gold/silver family assignment was based. When a sequence has been assigned to both a gold and silver standard family, this reference applies to both family classifications. When it has only been assigned to a silver standard family, this reference applies to the silver standard family classification. The full reference may be obtained by cross-referencing the ID number with Additional data file 4. Click here for file

Additional data file 2 Superfamily and family assignments for each of the sequences and structures from this work, as well as the corresponding Pfam and SCOP assignments. Pfam and SCOP assignments are listed as accession numbers. This file is essentially identical to Additional data file 1, except that Pfam and SCOP assignments are listed as database accession numbers rather than names, and SUPERFAMILY assignments are not listed. ^1^National Center for Biotechnology Information GI number. ^2^Protein Data Bank identifier. ^3^The gold and silver standard *o *-succinylbenzoate synthase (OSBS) families contain a more diverse set of enzymes than many other families listed in the table. All of the OSBS enzymes are believed to catalyze the same overall reaction via the same catalytic residues and there is no convincing evidence to suggest convergent evolution from within the superfamily, so we believe that these enzymes meet our definition of family. They appear, however, to utilize a different constellation of substrate binding residues, and certain subclusters within the family catalyze the promiscuous N-acyl amino acid racemase reaction in addition to the OSBS reaction. Because the sequences that comprise this family are highly divergent, it may pose special difficulties for automated clustering methods. Additional families that may be especially challenging include the extradiol dioxygenase families within the VOC superfamily, where a relatively high degree of sequence similarity and catalytic promiscuity make accurate clustering difficult. ^4^Evidence code for gold and silver standard family assignment [59]. ^5^ID number for the literature reference upon which gold/silver family assignment was based. When a sequence has been assigned to both a gold and silver standard family, this reference applies to both family classifications. When it has only been assigned to a silver standard family, this reference applies to the silver standard family classification. The full reference may be obtained by cross-referencing the ID number with Additional data file 4. Click here for file

Additional data file 3 Fasta format sequences for gold standard superfamily proteins. Some protein sequences will differ from the sequence listed for the equivalent GI number at the National Center for Biotechnology Information, as they have been trimmed to remove portions of the sequence that are not part of the superfamily. Click here for file

Additional data file 4 Literature references upon which gold and silver standard family assignments listed in Additional data file 1 and Additional data file 2 were made. Click here for file

Additional data file 5 Gold and silver standard family assignments for each of the sequences in this work, including the corresponding evidence codes and literature references. ^1^National Center for Biotechnology Information GI number. Additional data file 3 contains the fasta format sequences corresponding to each GI number. ^2^The gold and silver standard *o *-succinylbenzoate synthase (OSBS) families contain a more diverse set of enzymes than many other families listed in the table. All of the OSBS enzymes are believed to catalyze the same overall reaction via the same catalytic residues and there is no convincing evidence to suggest convergent evolution from within the superfamily, so we believe that these enzymes meet our definition of family. They appear, however, to utilize a different constellation of substrate binding residues, and certain subclusters within the family catalyze the promiscuous N-acyl amino acid racemase reaction in addition to the OSBS reaction. Because the sequences that comprise this family are highly divergent, it may pose special difficulties for automated clustering methods. Additional families that may be especially challenging include the extradiol dioxygenase families within the VOC superfamily, where a relatively high degree of sequence similarity and catalytic promiscuity make accurate clustering difficult. ^3^Evidence code for gold and silver standard family assignment [59]. ^4^ID number for the literature reference upon which gold/silver family assignment was based. This number corresponds to the reference ID number given in additional data files 1, 2, and 4. When a sequence has been assigned to both a gold and silver standard family, this reference applies to both family classifications. When it has only been assigned to a silver standard family, this reference applies to the silver standard family classification. Click here for file

## Figures and Tables

**Table 1:** Summary of gold standard superfamilies

Superfamily	Common chemical capability	Fold*	Number of families	Number of sequences^†^	Number of structures^‡^
Amidohydrolase	Metal ion(s) deprotonate water for nucleophilic attack on substrate	TIM beta/alpha-barrel	29	905	98
Crotonase	Stabilization of enolate anion intermediate derived from acyl-CoA substrate	ClpP/crotonase	16	970	22
Enolase	Abstraction of proton alpha to carboxylic acid, leading to a stabilized enolate anion intermediate	TIM beta/alpha-barrel	9	1,050	63
Haloacid dehalogenase	Active site Asp forms covalent enzyme-substrate intermediate, facilitating cleavage of C-Cl, P-C or P-O bond	HAD-like	20	1,281	50
Vicinal oxygen chelate	Metal coordination environment promotes direct electrophilic participation of metal in catalysis	Glyoxalase/bleomycin resistance protein/dihydroxybiphenyl dioxygenase	17	681	49

*Fold class, as defined by the Structural Classification of Proteins (SCOP). Note that the gold standard superfamilies are subsets of SCOP fold classes, and thus may not contain all members of their SCOP fold class. ^†^The number of sequences listed in this table for a gold standard superfamily may not match the corresponding number in the SFLD because some SFLD sequences are kept private, pending publication of the family into which they have been classified (these sequences appear in the gold standard set without a family classification), or because the SFLD may contain additional sequences obtained during periodic updating. ^‡^Includes mutant structures. Multiple structures may correspond to a single sequence.

**Table 2:** Summary of gold and silver standard families

Superfamily	Family	EC number*	Number of sequences (gold/silver)	Number of structures
Amidohydrolase	Aryldialkylphosphatase	3.1.8.1	2/3	0
	Phosphotriesterase	3.1.8.1	7/8	12
	Membrane dipeptidase	3.4.13.19	1/1	2
	N-acetylglucosamine-6-phosphate deacetylase	3.5.1.25	1/54	2
	Urease	3.5.1.5	100/107	35
	N-acyl-D-amino-acid deacylase	3.5.1.81	3/11	8
	D-hydantoinase	3.5.2.2	10/25	4
	L-hydantoinase	3.5.2.2	3/3	1
	Dihydroorotase1	3.5.2.3	3/79	0
	Dihydroorotase2	3.5.2.3	13/13	0
	Dihydroorotase3	3.5.2.3	7/43	1
	Allantoinase	3.5.2.5	4/7	0
	Imidazolonepropionase	3.5.2.7	1/29	0
	Cytosine deaminase	3.5.4.1	9/24	7
	Adenine deaminase	3.5.4.2	1/24	0
	Guanine deaminase	3.5.4.3	11/34	0
	Adenosine deaminase	3.5.4.4	10/20	17
	AMP deaminase	3.5.4.6	28/31	0
	Hydroxydechloroatrazine ethylaminohydrolase	3.5.99.3	1/2	0
	N-isopropylammelide isopropylaminohydrolase	3.5.99.4	1/1	0
	1-Aminocyclopropane-1-carboxylate deaminase	3.5.99.7	1/1	0
	Atrazine chlorohydrolase	3.8.1.8	1/1	0
	Glucuronate isomerase	5.3.1.12	1/2	1
	Ammelide aminohydrolase	NA	2/2	0
	Isoaspartyl dipeptidase	NA	5/5	5
	Melamine deaminase	NA	1/1	0
	N-acetylgalactosamine-6-phosphate deacetylase	NA	3/5	0
	S-triazine hydrolase	NA	1/1	0
	TrzN	NA	1/1	0
				
Crotonase	Histone acetyltransferase	2.3.1.48	11/12	0
	3-Hydroxyisobutyryl-CoA hydrolase	3.1.2.4	2/70	0
	4-Chlorobenzoate dehalogenase	3.8.1.7	1/7	2
	Methylmalonyl-CoA decarboxylase	4.1.1.41	1/6	2
	Cyclohexa-1,5-dienecarbonyl-CoA hydratase	4.2.1.100	1/3	0
	Enoyl-CoA hydratase	4.2.1.17	54/293	7
	Methylglutaconyl-CoA hydratase	4.2.1.18	2/5	1
	Methylglutaconyl-CoA hydratase 2	4.2.1.18	2/11	0
	Dodecenoyl-CoA delta-isomerase (mitochondrial)	5.3.3.8	2/13	1
	Dodecenoyl-CoA delta-isomerase (peroxisomal)	5.3.3.8	1/3	4
	Cyclohex-1-enecarboxyl-CoA hydratase	NA	1/2	0
	1,4-Dihydroxy-2-napthoyl-CoA synthase	NA	2/56	4
	2-Ketocyclohexanecarboxyl-CoA hydrolase	NA	1/1	0
	Crotonobetainyl-CoA hydratase	NA	2/15	0
	Delta(3,5)-delta(2,4)-dienoyl-CoA isomerase	NA	3/24	1
	Feruloyl-CoA hydratase/lyase	NA	5/18	0
				
Enolase	Enolase	4.2.1.11	215/375	20
	Glucarate dehydratase	4.2.1.40	26/31	7
	Galactonate dehydratase	4.2.1.6	5/27	0
	Methylaspartate ammonia-lyase	4.3.1.2	5/8	4
	Mandelate racemase	5.1.2.2	2/3	6
	Muconate cycloisomerase	5.5.1.1	14/26	5
	Chloromuconate cycloisomerase	5.5.1.7	10/15	3
	Dipeptide epimerase	NA	2/57	3
	Ortho-succinylbenzoate synthase	NA	6/75	4
				
Haloacid dehalogenase	Polynucleotide 5'-hydroxyl-kinase carboxy-terminal phosphatase	2.7.1.78	1/1	1
	Trehalose-phosphatase	3.1.3.12	1/2	0
	Histidinol-phosphatase	3.1.3.15	1/2	0
	Phosphoglycolate phosphatase	3.1.3.18	1/14	0
	Phosphoglycolate phosphatase 2	3.1.3.18	1/10	0
	Sucrose-phosphatase	3.1.3.24	5/13	0
	Phosphoserine phosphatase	3.1.3.3	2/56	9
	Deoxy-D-mannose-octulosonate 8-phosphate phosphatase	3.1.3.45	2/16	2
	5'-Nucleotidase	3.1.3.5	1/1	3
	2-Deoxyglucose-6-phosphatase	3.1.3.68	1/2	0
	Mannosyl-3-phosphoglycerate phosphatase	3.1.3.70	1/3	0
	Phosphonoacetaldehyde hydrolase	3.11.1.1	3/9	6
	P-type atpase	3.6.3.-	91/735	8
	2-Haloacid dehalogenase	3.8.1.2	7/20	8
	Beta-phosphoglucomutase	5.4.2.6	1/21	3
	Pyridoxal phosphatase	NA	1/1	0
	Enolase-phosphatase	NA	1/20	0
	Epoxide hydrolase N-terminal phosphatase	NA	2/2	6
	Glycerol-3-phosphate phosphatase	NA	1/3	0
	mdp-1	NA	1/2	2
				
Vicinal oxygen chelate	3,4-Dihydroxyphenylacetate 2,3-dioxygenase	1.13.11.15	4/9	6
	Catechol 2,3-dioxygenase	1.13.11.2	32/53	0
	4-Hydroxyphenylpyruvate dioxygenase	1.13.11.27	26/69	7
	2,3-Dihydroxybiphenyl dioxygenase	1.13.11.39	23/26	16
	4-Hydroxymandelate synthase	1.13.11.46	1/6	0
	Fosfomycin resistance protein FosA	2.5.1.18	2/4	6
	Glyoxalase I	4.4.1.5	12/58	9
	Methylmalonyl-CoA epimerase	5.1.99.1	5/9	2
	3-Methylcatechol 2,3-dioxygenase	NA	7/10	1
	2,6-Dichlorohydroquinone dioxygenase	NA	3/3	0
	2,3-Dihydroxy-p-cumate-3,4-dioxygenase	NA	2/3	0
	2,2',3-Trihydroxybiphenyl dioxygenase	NA	4/4	0
	1,2-Dihydroxynaphthalene dioxygenase	NA	6/17	0
	3-Isopropylcatechol-2,3-dioxygenase	NA	2/3	0
	2,4,5-Trihydroxytoluene oxygenase	NA	2/3	0
	Fosfomycin resistance protein FosB	NA	1/9	0
	Fosfomycin resistance protein FosX	NA	2/4	1

*Enzyme Commission Number for the primary reaction catalyzed by the family. Some families catalyze a characterized reaction for which no EC number has yet been assigned. The EC numbers for these families are designated as NA (not available).

**Table 3:** Comparison of gold and silver standard families to Pfam and SCOP families

Gold/silver standard family	Pfam family*	SCOP family*	Reaction catalyzed
Enolase	enolase_n, enolase_c	Enolase N-terminal domain-like, enolase	Dehydration of 2-phospho-D-glycerate
Methylaspartate ammonia-lyase	maal_n, maal_c	Enolase N-terminal domain-like, D-glucarate dehydratase-like	Elimination of ammonia from methylaspartic acid
Mandelate racemase	mr_mle_n, mr_mle	Enolase N-terminal domain-like, D-glucarate dehydratase-like	Racemization of S-mandelate to R-mandelate
Dipeptide epimerase	mr_mle_n, mr_mle	Enolase N-terminal domain-like, D-glucarate dehydratase-like	Dipeptide epimerization
Chloromuconate cycloisomerase	mr_mle_n, mr_mle	Enolase N-terminal domain-like, D-glucarate dehydratase-like	Chloromuconate lactonization
Muconate cycloisomerase	mr_mle_n, mr_mle	Enolase N-terminal domain-like, D-glucarate dehydratase-like	Muconate lactonization
Ortho-succinylbenzoate synthase	mr_mle_n, mr_mle	Enolase N-terminal domain-like, D-glucarate dehydratase-like	Dehydration of 2-succinyl-6-hydroxy-2,4-cyclohexadiene-1-carboxylic acid
Glucarate dehydratase	mr_mle_n, mr_mle	Enolase N-terminal domain-like, D-glucarate dehydratase-like	Dehydration of D-glucarate
Galactonate dehydratase	mr_mle_n, mr_mle	NA	Dehydration of D-galactonate
Fosfomycin resistance protein FosA	Glyoxalase	Antibiotic resistance proteins	Addition of glutathione to the oxirane ring of fosfomycin
2,3-Dihydroxybiphenyl dioxygenase	Glyoxalase	Extradiol dioxygenases	Extradiol cleavage of 2,3-dihydroxybiphenyl to 2-hydroxy-6-oxo-6-phenylhexa-2,4-dienoate
3,4-Dihydroxyphenylacetate 2,3-dioxygenase	Glyoxalase	Extradiol dioxygenases	extradiol cleavage of 3,4-dihydroxyphenylacetate to 2-hydroxy-5-carboxymethylmuconate semialdehyde
3-Methylcatechol 2,3-dioxygenase	Glyoxalase	Extradiol dioxygenases	Extradiol cleavage of 3-methylcatechol to 2-hydroxy-6-oxo-2,4-heptadienoate
4-Hydroxyphenylpyruvate dioxygenase	Glyoxalase	Extradiol dioxygenases	Conversion of 4-hydroxyphenylpyruvate to homogentisate
Glyoxalase I	Glyoxalase	Glyoxalase I	Conversion of methylglyoxal (hemithioacetal form) to S-D-lactoylglutathione
Methylmalonyl-CoA epimerase	Glyoxalase	Methylmalonyl-CoA epimerase	Epimerization of (2R)-methylmalonyl-CoA to (2S)-methylmalonyl-CoA
1,2-Dihydroxynaphthalene dioxygenase	Glyoxalase	NA	Extradiol cleavage of 1,2-dihydroxynaphthalene
2,2',3-Trihydroxybiphenyl dioxygenase	Glyoxalase	NA	Extradiol cleavage of 2,2',3-trihydroxybiphenyl to 2-hydroxy-6-oxo-(2-hydroxyphenyl)-hexa-2,4-dienoic acid
2,3-Dihydroxy-p-cumate-3,4-dioxygenase	Glyoxalase	NA	Extradiol cleavage of 2,3-dihydroxy-p-cumate to 2-hydroxy-3-carboxy-6-oxo-7-methylocta-2,4-dienoate
2,4,5-Trihydroxytoluene oxygenase	Glyoxalase	NA	Extradiol cleavage of 2,4,5-trihydroxytoluene
2,6-Dichlorohydroquinone dioxygenase	Glyoxalase	NA	Extradiol cleavage of 2,6-dichlorohydroquinone
3-Isopropylcatechol-2,3-dioxygenase	Glyoxalase	NA	Extradiol cleavage of 3-isopropylcatechol
4-Hydroxymandelate synthase	Glyoxalase	NA	Conversion of p-hydroxyphenylpyruvate to L-p-hydroxymandelate
Catechol 2,3-dioxygenase	Glyoxalase	NA	Extradiol cleavage of catechol to alpha-hydroxymuconic semialdehyde
Fosfomycin resistance protein FosB	Glyoxalase	NA	Addition of L-cysteine to the oxirane ring of fosfomycin
Fosfomycin resistance protein FosX	Glyoxalase	NA	Addition of water to the oxirane ring of fosfomycin
Adenosine deaminase	A_deaminase	Adenosine deaminase (ADA)	Deamination of adenosine
AMP deaminase	A_deaminase	NA	Deamination of AMP
Cytosine deaminase	Amidohydro_1	Cytosine deaminase catalytic domain; cytosine deaminase	Deamination of cytosine
N-acyl-D-amino-acid deacylase	Amidohydro_1	D-aminoacylase, catalytic domain; D-aminoacylase	Hydrolysis of an N-acyl-D-amino-acid
Dihydroorotase3	Amidohydro_1	Dihydroorotase	Synthesis of dihydroorotate from carbamoyl aspartate
D-hydantoinase	Amidohydro_1	Hydantoinase (dihydropyrimidinase), catalytic domain; hydantoinase (dihydropyrimidinase)	Hydrolytic ring cleavage of a dihydropyrimidine
L-hydantoinase	Amidohydro_1	Hydantoinase (dihydropyrimidinase), catalytic domain; hydantoinase (dihydropyrimidinase)	Hydrolytic ring cleavage of a 5 membered cyclic diamide
Isoaspartyl dipeptidase	Amidohydro_1	Isoaspartyl dipeptidase, catalytic domain; isoaspartyl dipeptidase	Hydrolysis of beta-l-isoaspartyl linkage of a dipeptide
Adenine deaminase	Amidohydro_1	NA	Deamination of adenine
Allantoinase	Amidohydro_1	NA	Hydrolysis of allantoin
Ammelide aminohydrolase	Amidohydro_1	NA	Deamination of ammelide
Aryldialkylphosphatase	Amidohydro_1	NA	Hydrolysis of an organophosphate
Atrazine chlorohydrolase	Amidohydro_1	NA	Hydrolytic dechlorination of atrazine
Dihydroorotase1	Amidohydro_1	NA	Synthesis of dihydroorotate from carbamoyl aspartate
Dihydroorotase2	Amidohydro_1	NA	Synthesis of dihydroorotate from carbamoyl aspartate
Guanine deaminase	Amidohydro_1	NA	Deamination of guanine
Hydroxydechloroatrazine ethylaminohydrolase	Amidohydro_1	NA	Conversion of 4-(ethylamino)-2-hydroxy-6-(isopropylamino)-1,3,5-triazine to N-isopropylammelide
Imidazolonepropionase	Amidohydro_1	NA	Hydrolysis of (S)-3-(5-oxo-4,5-dihydro-3H-imidazol-4-yl)propanoate
Melamine deaminase	Amidohydro_1	NA	Deamination of melamine
N-acetylgalactosamine-6-phosphate deacetylase	Amidohydro_1	NA	Deacetylation of N-acetylgalactosamine-6-phosphate
N-isopropylammelide isopropylaminohydrolase	Amidohydro_1	NA	Conversion of N-isopropylammelide to isopropylamine
S-triazine hydrolase	Amidohydro_1	NA	Hydrolysis of a triazine
Trzn	Amidohydro_1	NA	Hydrolysis of a triazine
N-acetylglucosamine-6-phosphate deacetylase	Amidohydro_1	N-acetylglucosamine-6-phosphate deacetylase, catalytic domain; N-acetylglucosamine-6-phosphate deacetylase	Deacetylation of N-acetylglucosamine-6-phosphate
Urease	Amidohydro_1, urease	Alpha-subunit of urease, catalytic domain; alpha-subunit of urease; urease, beta-subunit; urease, gamma-subunit	Hydrolysis of urea to ammonia and carbon dioxide
1-Aminocyclopropane-1-carboxylate deaminase	None	NA	Deamination of 1-aminocyclopropane-1-carboxylate
Phosphotriesterase	PTE	Phosphotriesterase-like	Hydrolysis of an organophosphate
Membrane dipeptidase	Renal_dipeptase	Renal dipeptidase	Hydrolysis of a dipeptide
Glucuronate isomerase	UxaC	Uronate isomerase TM0064	Conversion of D-glucuronate to D-frucuronate
Delta(3,5)-delta(2,4)-dienoyl-CoA isomerase	ECH	Crotonase-like	Isomerization of 3,5-dienoyl-CoA to 2,4-dienoyl-CoA
Methylmalonyl-CoA decarboxylase	ECH	Crotonase-like	Decarboxylation of methylmalonyl CoA
Methylglutaconyl-CoA hydratase	ECH	Crotonase-like	Hydration of 3-methylglutaconyl-CoA
Enoyl-CoA hydratase	ECH	Crotonase-like	Hydration of trans-2-enoyl-CoA thiolester
4-Chlorobenzoate dehalogenase	ECH	Crotonase-like	Hydrolytic dehalogenation of 4-chlorobenzoyl-CoA
Dodecenoyl-CoA delta-isomerase (peroxisomal)	ECH	Crotonase-like	Isomerization of 3-enoyl-CoA to 2-enoyl-CoA
Methylglutaconyl-CoA hydratase 2	ECH	NA	Hydration of 3-methylglutaconyl-CoA
Histone acetyltransferase	ECH	NA	Acetylation of histone
2-Ketocyclohexanecarboxyl-CoA hydrolase	ECH	NA	Cleavage of 2-ketocyclohexanecarboxyl-CoA to pimelyl-CoA
1,4-Dihydroxy-2-napthoyl-CoA synthase	ECH	NA	Cyclization of *o *-succinylbenzoate-CoA thioester
Feruloyl-CoA hydratase/lyase	ECH	NA	Hydration and nonoxidative cleavage of feruloyl-SCoA to vanillin and acetyl-SCoA
Crotonobetainyl-CoA hydratase	ECH	NA	Hydration of crotonobetainyl-CoA
Cyclohex-1-enecarboxyl-CoA hydratase	ECH	NA	Hydration of cyclohex-1-enecarboxyl-CoA
Cyclohexa-1,5-dienecarbonyl-CoA hydratase	ECH	NA	Hydration of cyclohexa-1,5-diene-1-carboxyl-CoA
3-Hydroxyisobutyryl-CoA hydrolase	ECH	NA	Hydrolysis of 3-hydroxyisobutyryl-CoA
Dodecenoyl-CoA delta-isomerase (mitochondrial)	ECH	NA	Isomerization of 3-enoyl-CoA to 2-enoyl-CoA
Beta-phosphoglucomutase	Hydrolase	Beta-phosphoglucomutase-like	Conversion of beta-glucose-1-phosphate to glucose-6-phosphate
P-type ATPase	Hydrolase	Calcium ATPase, catalytic domain P	Dephosphorylation of ATP to ADP
Epoxide hydrolase N-terminal phosphatase	Hydrolase	Epoxide hydrolase, N-terminal domain	Dephosphorylation
2-Haloacid dehalogenase	Hydrolase	L-2-haloacid dehalogenase, HAD	Dehalogenation of (s)-2-haloacid
Phosphoserine phosphatase	Hydrolase	Phosphoserine phosphatase	Dephosphorylation of phosphoserine
Phosphonoacetaldehyde hydrolase	Hydrolase	Phosphonoacetaldehyde hydrolase	Hydrolysis of phosphonoacetaldehyde
2-Deoxyglucose-6-phosphatase	Hydrolase	NA	Dephosphorylation of 2-deoxyglucose-6-phosphate
Phosphoglycolate phosphatase	Hydrolase	NA	Dephosphorylation of 2-phosphoglycolate
Phosphoglycolate phosphatase 2	Hydrolase	NA	Dephosphorylation of 2-phosphoglycolate
Glycerol-3-phosphate phosphatase	Hydrolase	NA	Dephosphorylation of glycerol-3-phosphate
Pyridoxal phosphatase	Hydrolase	NA	Dephosphorylation of pyridoxal 5'-phosphate
Enolase-phosphatase	Hydrolase	NA	Oxidative cleavage
Histidinol-phosphatase	IGPD	NA	Dephosphorylation of L-histidinol-phosphate
Sucrose-phosphatase	S6PP	NA	Dephosphorylation of sucrose 6-phosphate
Trehalose-phosphatase	Trehalose_ PPase	NA	Dephosphorylation of trehalose 6-phosphate
5'-Nucleotidase	None	5' (3')-Deoxyribonucleotidase (dNT-2)	Dephosphorylation of 5' nucleotide
Deoxy-D-mannose-octulosonate 8-phosphate phosphatase	None	Probable phosphatase YrbI	Dephosphorylation of 3-deoxy-D-manno-octulosonate 8-phosphate
Polynucleotide 5'-hydroxyl-kinase carboxy-terminal phosphatase	None	Polynucleotide kinase, phosphatase domain	Dephosphorylation of 3' nucleotide
mdp-1	None	NA	Dephosphorylation
Mannosyl-3-phosphoglycerate phosphatase	None	NA	Dephosphorylation of 2(alpha-D-mannosyl)-3-phosphoglycerate

*Some gold standard families correspond to multiple Pfam and/or SCOP families because Pfam and SCOP divide the enzymes in question into multiple structural domains, each with a different family assignment. NA = Not applicable, IGPD, Pfam Imidazoleglycerol-phosphate dehydratase family; ECH, Pfam Enoyl-CoA hydratase/isomerase family; PTE, Pfam Phosphotriesterase family.
